# A Critical Review of Alberta Stroke Program Early CT Score for Evaluation of Acute Stroke Imaging

**DOI:** 10.3389/fneur.2016.00245

**Published:** 2017-01-12

**Authors:** Julian Schröder, Götz Thomalla

**Affiliations:** ^1^Department of Neurology, University Medical Center Hamburg-Eppendorf, Hamburg, Germany

**Keywords:** stroke, acute stroke treatment, computed tomography, magnetic resonance imaging, Alberta Stroke Program Early CT Score

## Abstract

Assessment of ischemic stroke lesions on computed tomography (CT) or MRI using the Alberta Stroke Program Early CT Score (ASPECTS) is widely used to guide acute stroke treatment. We aimed to review the current evidence on ASPECTS. Originally, the score was developed for standardized lesion assessment on non-contrast CT (NCCT). Early studies described ASPECTS as a predictor of functional outcome and symptomatic intracranial hemorrhage after iv-thrombolysis with a threshold of ≤7 suggested to identify patients at high risk. Following studies rather pointed toward a linear relationship between ASPECTS and functional outcome. ASPECTS has also been applied to assess perfusion CT and diffusion-weighted MRI (DWI). Cerebral blood volume ASPECTS proved to be the best predictor of outcome, outperforming NCCT-ASPECTS in some studies. For DWI-ASPECTS varying thresholds to identify patients at risk for poor outcome were reported. ASPECTS has been used for patient selection in three of the five groundbreaking trials proving efficacy of mechanical thrombectomy published in 2015. ASPECTS values predict functional outcome after thrombectomy. Moreover, treatment effect of thrombectomy appears to depend on ASPECTS values being smaller or not present in low ASPECTS, while patients with ASPECTS 5–10 do clearly benefit from mechanical thrombectomy. However, as patients with low ASPECTS values were excluded from recent trials data on this subgroup is limited. There are several limitations to ASPECTS addressed in a growing number of studies. The score is limited to the anterior circulation, the template is unequally weighed and correlation with lesion volume depends on lesion location. Overall ASPECTS is a useful and easily applicable tool for assessment of prognosis in acute stroke treatment and to help guide acute treatment decisions regardless whether MRI or CT is used. Patients with low ASPECTS values are unlikely to achieve good outcome. However, methodological constraints of ASPECTS have to be considered, and based on present data, a clear cutoff value to define “low ASPECTS values” cannot be given.

## Introduction

The Alberta Stroke Program Early Computed Tomography Score (ASPECTS) is widely used in clinical practice to assess the extent of early ischemic changes on brain imaging for acute stroke treatment. ASPECTS has been applied to various imaging modalities in acute stroke imaging since its introduction in 2000. ASPECTS is a 10-point scoring system with anatomical regions distributed over the MCA territory (1).

It was designed as a robust imaging measure to predict outcome in intravenous thrombolysis. ASPECTS has drawn a lot of attention due to its use for patient exclusion in the 2015 trials demonstrating efficacy of mechanical thrombectomy (2–4).

**Figure 1:**
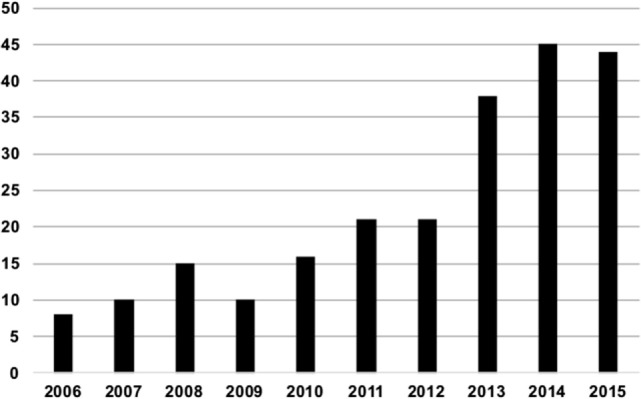
**Number of publications indexed in MedLine for the search term “Alberta Stroke Program early CT Score” per year since 2006**.

Due to high efficacy, we will see an increase of mechanical thrombectomy over the course of the next years and with it probably an increasing use of ASPECTS in routine clinical practice, as patient stratification is key in this time-dependent treatment. There are also a rapidly growing number of scientific studies using ASPECTS in stroke research or addressing methodological questions concerning ASPECTS (please see Figure 1 for an overview of the number of studies published per year over the last 10 years). This article aims to summarize the current evidence on ASPECTS in a topical and selective review and to explain its applications in clinical practice and trials.

## Original CT Score

The ECASS-1 trial first established the relevance of early ischemic signs on non-contrast CT scans prior to intravenous thrombolysis (5). Von Kummer et al. showed in 1997 that patients with early ischemic changes in over one-third of the MCA territory had a lower chance of good outcome after iv-rtPA (6). However, identification of patients following the 1/3 of the MCA territory paradigm proved to be unreliable in clinical practice (7).

Given these limitations to the 1/3 rule and the necessity to assess early ischemic changes in a reliable way, Barber et al. developed ASPECTS. The score was intended as a pragmatic, reliable, and easily applicable scoring template for early ischemic changes on CT (1).

**Figure 2:**
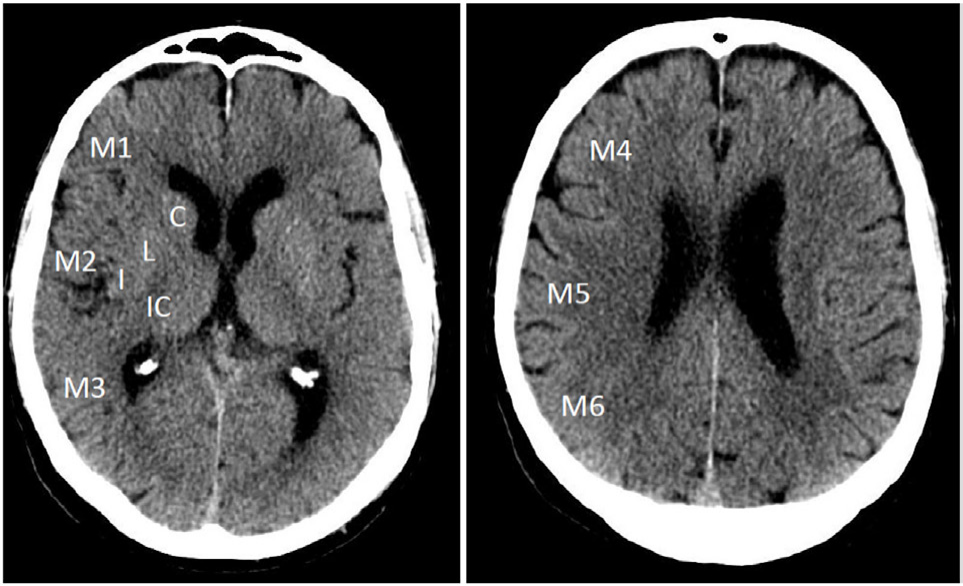
**Alberta Stroke Program Early Computed Tomography Score template on non-contrast CT with 10 regions distributed over the MCA territory in ganglionic and supraganglionic levels**.

The template consists of 10 anatomically defined regions, 4 for subcortical structures [caudate (C); lentiform (L); internal capsule (IC); insular ribbon (I)] and 6 for cortical structures in the MCA territory, labeled M1–M6 (1, 8) (Figure 2).

The MCA territory is assessed on all axial CT cuts in two distinct levels, the “ganglionic” and “supraganglionic” level. All axial cuts on the level of the caudate head or below are hereby allotted to the ganglionic level, all above to the supraganglionic. The caudate nucleus is part of both layers, the head belonging to the ganglionic, body, and tail to the supraganglionic level (8).

Early ischemic changes on CT were originally defined as intraparenchymal hypoattenuation (loss of gray–white matter distinction) and focal swelling. For each ASPECTS region that presents with early ischemic changes on at least two consecutive cuts, the overall score of 10 is reduced by 1. Thus, a score of 0 would indicate infarction of all 10 regions.

Despite relatively vague definition of the individual regions interrater agreement for dichotomized ASPECTS has been described as good (1, 9, 10) with some studies reporting moderate agreement (11–13).

## ASPECTS as Predictor of Stroke Outcome

The original publication proposed a cutoff of ≤7 on the initial non-contrast CT (NCCT) as it predicted functional dependence in patients who underwent thrombolysis within 3 h from symptom onset (1).

A larger Canadian study of 936 patients treated with iv-thrombolysis in 3 h time window between 1999 and 2001 demonstrated a near linear inverse relationship between ASPECTS on baseline NCCT and functional outcome (14). However, in 2012, González et al. reported no significant prediction of MRS >2 after 6 months by ASPECTS on initial NCCT performed within 24 h from symptom onset in 649 patients diagnosed with ischemic stroke (15).

For iv-thrombolysis a modification of treatment effect by ASPECTS could not be proven in 3 or 6 h time windows (16, 17). Still, in the NINDS rtPA Stroke Study higher ASPECTS values were associated with a greater benefit from iv treatment with rtPA (16).

## ASPECTS as Predictor of Symptomatic Intracranial Hemorrhage after Thrombolysis

Barber et al. originally described ASPECTS as a significant predictor of symptomatic intracranial hemorrhage after thrombolysis within 3 h from symptom onset (1). This could not be reproduced based on data from the ECASS-II or NINDS-stroke trials (16, 17).

In 2009, Puetz et al. published a comprehensive review focusing mainly on NCCT-ASPECTS. As isolated cortical swelling can occur in penumbra and infarct core, they proposed the removal of isolated focal swelling without hypoattenuation from the early ischemic changes relevant for scoring (18).

## Use of ASPECTS with Perfusion-CT and Diffusion-Weighted MRI

There have been numerous publications assessing the applicability of ASPECTS to multiparametric CT and MRI. One prominent focus has been the evaluation of different CT Perfusion measures using ASPECTS.

Parsons et al. described cerebral blood volume (CBV) ASPECTS within 6 h from symptom onset as a more accurate predictor of irreversibly damaged tissue when compared with NCCT ASPECTS in 2007 (19). Lin et al. identified a MTT/CBV ASPECTS mismatch of ≥1 within 6 h as the optimal cutoff to identify a volumetric mismatch of ≥20% (20). MTT/CBV ASPECTS mismatch within 3 h from symptom onset was also highly correlated with volumetric tissue at risk in a 2011 study by Sillanpaa et al.; furthermore, CBV ASPECTS within 8 h from symptom onset was superior to NCCT ASPECTS in discriminating patients with favorable outcome (21, 22). In contrast, a large study with 824 patients from a Dutch stroke registry could not find an additional impact of CBV and MTT ASPECTS compared to only NCCT ASPECTS within 9 h from symptom onset when analyzed in a multivariate model. In the same study, CBV and MTT ASPECTS were significant predictors of poor clinical outcome in univariate analysis (23).

Thus, it is unclear whether CTP ASPECTS offers a clear advantage over NCCT ASPECTS, data at the present state are ambiguous. There have been reports of improved interrater reliability on CBV ASPECTS within 4.5 and 9 h from stroke onset compared to NCCT ASPECTS (13, 24).

ASPECTS has also been used to assess lesion extent on diffusion-weighted MRI (DWI), usually labeled “DWI-ASPECTS.” DWI-ASPECTS within 3 h after symptom onset has been proven to predict functional outcome (MRS) and mortality after 3 months in patients undergoing iv-thrombolysis. As a cutoff to identify patients at risk for poor outcome DWI-ASPECTS >6 was proposed, though specificity was low at 33% (25). Another study identified DWI-ASPECTS >5 within 3 h after onset as a cutoff to identify patients with good functional outcome 7 days after iv-thrombolysis (26). Based on a cohort of patients with imaging between 3 and 24 h after onset, Tei et al. proposed a cutoff of DWI-ASPECTS >7 to predict MRS ≥3 after 3 months in 2011 (27). In all three studies, DWI-ASPECTS was an independent predictor of functional outcome. Nezu et al. found DWI-ASPECTS to be approximately 1 point lower than NCCT-ASPECTS within 3 h from symptom onset in 360 patients, who underwent both imaging modalities. In the same study, there was no significant difference in area under the receiver operating characteristic (ROC) curve of DWI- and NCCT-ASPECTS for prediction of MRS 0–2 at 90 days. Interrater agreement was higher for DWI-ASPECTS (28).

Overall due to the higher sensitivity of DWI (29), DWI-ASPECTS is more sensitive for the detection of early ischemic changes than NCCT ASPECTS (30). After endovascular therapy within 12 h from symptom onset DWI-ASPECTS had higher interrater agreement and according to ROC analysis outperformed NCCT ASPECTS in predicting good functional outcome at 90 days poststroke (9).

There have been multiple attempts to use ASPECTS as a surrogate marker for DWI lesion volume and a threshold of <4 has been proposed to identify patients with DWI volume >100 ml (31, 32). We could show that depending on lesion location estimation of DWI lesion volume by DWI-ASPECTS is unreliable (see below) (33).

## ASPECTS for Use with Mechanical Thrombectomy

Identification of a clearly defined treatable lesion was highlighted as a key issue (34, 35) after three endovascular trials published in 2013 (36–38) failed to prove a significant additional benefit of endovascular stroke treatment over standard iv treatment. As the acute stroke setting comes along with restrictions for time consuming post processing of CT or MRI data, ASPECTS among other techniques has been proposed as a fast and easy method to identify patients suitable for endovascular reperfusion treatment.

There have been several studies suggesting an increased benefit of endovascular treatment for patients with higher ASPECTS values using various imaging modalities.

In the Penumbra Pivotal Stroke (39) trial not a single patient with ASPECTS <5 on the initial CT scan up to 8 h after symptom onset achieved a favorable outcome (MRS 0–2 after 3 months), good outcome was significantly more frequent in the ASPECTS score >7 group when compared to the ASPECTS score ≤7 group [50 vs. 15%; RR, 3.3; 95% confidence interval (CI), 1.6–6.8; *p* < 0.0001].

A further analysis of data from trials using the Penumbra system including 249 patients again showed an increase in favorable outcome with higher ASPECTS values (40). Symptomatic intracranial hemorrhage was more frequent in patients with low ASPECTS scores on initial CT scans. Furthermore, patients with ASPECTS 0–4 had significantly worse outcome than patients with ASPECTS 5–10 and did not benefit from faster treatment, thus suggesting a cutoff ASPECTS ≤4 to identify patients with poor response to intravascular treatment (40).

In a study of 51 patients undergoing aspiration thrombectomy with a median onset to recanalization time of 292 (IQR 246–357) min, patients with good outcome (MRS 0–2) had significantly higher CBV ASPECTS (CBV ASPECTS 8 vs. 6, *p* = 0.0007), CBV ASPECTS >7 was identified as optimal cutoff with a positive predictive value of 65% (41). This was reproduced by Lum et al. in a collective of 46 patients within 6 h from symptom onset (42).

Soize et al. reported an independent prediction of outcome and of symptomatic intracranial hemorrhage by DWI-ASPECTS in 59 patients after mechanical thrombectomy (mean time from symptom onset to recanalization 296 min) (43). Inoue et al. identified DWI-ASPECTS ≥5 as the optimal predictor of favorable outcome after 90 days following intra-arterial treatment in a collective of 210 patients [median time from onset to MRI 105 (IQR 75–178) min] (44).

In the IMS-III trial (656 subjects randomized), patients with ASPECTS 8–10 on initial NCCT up to 3 h after symptom onset were almost twice as likely (relative risk, 1.8; 99% CI, 1.4–2.4) to achieve a favorable outcome. However, there was no significant treatment by ASPECTS interaction (45).

## ASPECTS in the Large Stent-Triever Thrombectomy Trials

In 2015, five randomized-controlled trials demonstrated a strong positive effect of mechanical thrombectomy using stent-triever devices on patient outcome when compared with standard iv treatment alone. Most of these trials used NCCT-ASPECTS for patient selection based on the experience of the studies cited above.

The first new generation thrombectomy trial published, MR CLEAN did not use an ASPECTS threshold for patient exclusion. Patients were included up to 6 h after symptom onset. There was a consistent additional effect of intra-arterial treatment over all ASPECTS ranges analyzed (0–4, 5–7, 8–10). Intra-arterial treatment caused no increase of symptomatic intracranial hemorrhage in any of the ASPECTS groups compared iv treatment only. However, only 30 patients with ASPECTS 0–4 were analyzed and only one of those achieved MRS 2 (46, 47).

Based on the findings of Inoue et al. (44), the SWIFT-PRIME study used NCCT- or DWI-ASPECTS ≤5 within 6 h after symptom onset as an exclusion threshold. There was no difference in outcome between patients with ASPECTS 6–7 and 8–10 (4). Investigators in the ESCAPE study also applied an ASPECTS threshold of ≤5 up to 12 h after symptom onset to exclude patients. Again, there was no heterogeneity of effect between patients with ASPECTS 6–7 and 8–10 (2).

The only study to use DWI-ASPECTS and NCCT-ASPECTS in a time window up to 8 h after symptom onset was REVASCAT. To account for the different sensitivities of DWI and NCCT-imaging a threshold of <7 was applied to CT and <6 to DWI for patient exclusion. The difference of 1 point between DWI- and NCCT-ASPECTS was chosen based on earlier findings by Nezu et al. (28). There was no significant difference in outcome for patients with ASPECTS ≤7 and above (3). In EXTEND-IA, patient selection was not based on ASPECTS but on volumetric infarct core to perfusion lesion mismatch (48).

**Figure 3:**
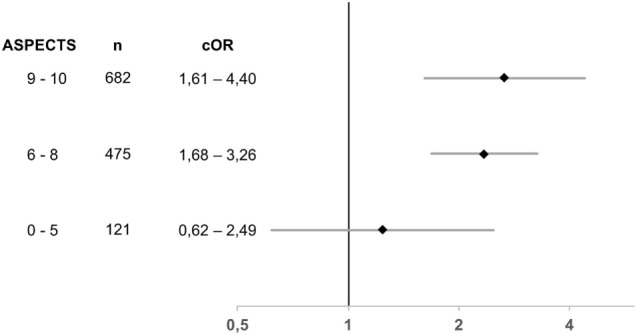
**Odds ratios for adjusted treatment effect for MRS 0–2 at 90 days stratified for different Alberta Stroke Program Early Computed Tomography Score subgroups in the HERMES meta-analysis; there was no significant heterogeneity of effect (*p* = 0.29); *n* indicates the number of patients analyzed; cOR, common odds ratio**. Based on data from Goyal et al. (49).

A recent meta-analysis performed a central reading of all pre-treatment scans from the five thrombectomy trials and found a clear benefit of thrombectomy in patients with ASPECTS >5 (49). Of note, in the pooled data, median ASPECTS was 9 for both the treatment (IQR 7–10) and control (IQR 8–10) groups. Treatment effect was analyzed for three ASPECTS strata: 0–5, 6–8, and 9–10. While there was a strong and consistent treatment effect for both ASPECTS 6–8 and 9–10 with an adjusted odds ratio of OR 2.36 (95% CI 1.68–3.26) and 2.66 (1.61–4.40), no clear benefit was observed for 121 patients with ASPECT 0–5 with an OR of 1.24 (0.62–2.42). Figure 3 illustrates the odds ratios for the different ASPECTS subgroups.

## Methodological Issues and Limitations of ASPECTS

Despite its broad application ASPECTS has limitations. First of all, the original ASPECTS score is limited to the anterior circulation only (1). Second, ASPECTS shows an unequal weighing of brain regions, as first described in 2006 by Phan et al. (50). The template is based on anatomical structures, and thus, the individual regions cover different amounts of brain tissue. Additionally, the exact extent of each region or how much damaged tissue is required to render a region affected has never been defined (1).

**Figure 4:**
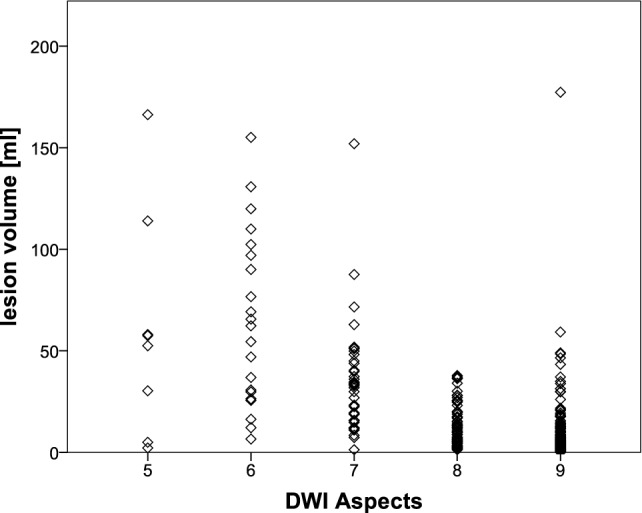
**Distribution of lesion volumes in patients with diffusion-weighted MRI (DWI)-Alberta Stroke Program Early Computed Tomography Score (ASPECTS) ≥5, these patients were shown to benefit from mechanical thrombectomy in the recent HERMES meta-analysis (49)**. Lesion volumes varied considerably in patients with the same DWI ASPECTS. Based on data from Schröder et al. (33).

In a study of 496 patients, we could show that correlation of ASPECTS with DWI lesion volume varied considerably depending on lesion location (33). Figure 4 shows the distribution of lesion volumes for ASPECTS values >4. Two patients with the same ASPECTS score do not necessarily have similar lesion volumes. As lesion volume is a strong predictor of functional outcome (51–53), the template’s unequal weighing could compromise clinical decisions. If decision making is based solely on an ASPECTS threshold this might lead to unjustified exclusion of patients from clinical trials or even treatment when lesion location is not considered.

Depending on analyzed sample, treatment, and imaging modality applied, various cutoffs to identify patients at risk of poor outcome have been suggested (1, 14, 20, 25–27, 47). Furthermore, large studies suggested a linear relationship or even no significant outcome prediction by ASPECTS at all (14, 15). Thus, recommendation of one single threshold to identify patients with poor outcome based on the available data seems hardly justifiable.

Another issue confined to the widely used NCCT ASPECTS is the poor sensitivity in the early period after stroke (29).

## Conclusion

ASPECTS is a useful and easily applicable tool for standardized evaluation of the extent of acute ischemic lesions that may help in the assessment of prognosis in acute stroke treatment regardless whether MRI or CT is used. Patients with low ASPECTS values are unlikely to achieve good outcome. However, based on present data, a clear cutoff value to define “low ASPECTS values” cannot be given.

A clear advantage of CT perfusion ASPECTS over NCCT ASPECTS has not been established, both could be shown to predict poor functional outcome as assessed by the MRS after 90 days. CBV ASPECTS may offer slightly improved interrater reliability.

Due to DWI’s high sensitivity, a key issue of DWI-ASPECTS is the definition how much lesioned tissue is required for a region to be counted as affected. Formal instructions are lacking. This could explain at least part of the variance in the proposed cutoff values. There are contradicting reports comparing the performances of DWI-ASPECTS and NCCT-ASPECTS for outcome prediction. Interrater agreement was improved for DWI-ASPECTS compared to NCCT-ASPECTS.

ASPECTS may further helpful in guiding patient selection for enrollment in clinical trials as well as for reperfusion treatment. For mechanical thrombectomy, a clear benefit over iv treatment alone has been proven for patients with NCCT ASPECTS 6–10, while for ASPECTS values 0–5 treatment effect is not clear.

However, when applying ASPECTS cutoffs methodological limitations have to be considered resulting from the unequal weighing of different brain regions by ASPECTS. As a consequence, stroke lesions with the same ASPECTS rating may have quite different lesion extent depending on their location. Thus, we do not recommend to stick to strict NCCT- or DWI-ASPECTS values for exclusion of patients from treatment but rather consider ASPECTS as a helpful of diagnostic tools that should be looked at in the broader perspective including other imaging and clinical features.

## Author Contributions

Both JS and GT made substantial contributions to the conception, the acquisition, analysis, and interpretation of data for the work; drafted the work and/or revised it critically for important intellectual content; approved the final version to be published; and agreed to be accountable for all aspects of the work in ensuring that questions related to the accuracy or integrity of any part of the work are appropriately investigated and resolved.

## Conflict of Interest Statement

The authors declare that the research was conducted in the absence of any commercial or financial relationships that could be construed as a potential conflict of interest.
